# Retraction: Sarcosine Up-Regulates Expression of Genes Involved in Cell Cycle Progression of Metastatic Models of Prostate Cancer

**DOI:** 10.1371/journal.pone.0268981

**Published:** 2022-05-19

**Authors:** The PLOS ONE Editors

Following the publication of this article [1], the corresponding author informed the journal of the following issues in Figs 1 and 4:

- In Fig 1B, the PC-3 0 h Sarcosine+ panel partially overlaps with the LNCaP 0 h Sarcosine–.
- In Fig 4B, the following panels appear to partially or fully overlap:
  ○ PC-3 *KNTC2* and LNCaP *AR* panels.
  ○ PC-3 *CCND1* and LNCaP *KLK4* panels.
  ○ PC-3 *IVNS1ABP* and LNCaP *RCN1* panels.
  ○ PC-3 *MAPK8* and *KLK3* panels.
  ○ PC-3 *β-actin* panel and LNCaP *β-actin* panel flipped horizontally and vertically.

The corresponding author indicated that the raw data underling the results reported in all figures other than Fig 4B and the Supporting Information files are available.

The corresponding author stated that an error occurred in the preparation of Fig 1B. They provided a replacement image for the LNCaP 0 h Sarcosine–panel, and raw data underlying the results in Fig 1B. The journal considers the concerns with this figure resolved.

The corresponding author provided unannotated images described as raw data underlying Fig 4B. They acknowledged that they were unable to accurately annotate the raw image data as the relevant laboratory records are incomplete. They requested that the article be retracted because the issues with the published figures cannot be fully resolved.

In light of the concerns affecting Fig 4B that question the integrity of these data, and in the absence of raw underlying data, the *PLOS ONE* Editors retract this article.

ZH, DP, TE and VA agreed with the retraction. ZH and VA stand by the article’s findings and apologize for the issues with the published article. MAMR, PM, HP, MM, VV and MP either did not respond directly or could not be reached. MS is deceased.
